# Gut Microbial Functional Ecology and Microbiota-Derived Metabolites in Rheumatoid Arthritis Autoimmunity

**DOI:** 10.3390/cells15151398

**Published:** 2026-08-01

**Authors:** Xinqian Rong, Xiaomeng Zhang, Yong Tan, Cheng Lu

**Affiliations:** Institute of Basic Research in Clinical Medicine, China Academy of Chinese Medical Sciences, Beijing 100700, China; rongxinqian2016@163.com (X.R.); zhxmzoe@163.com (X.Z.)

**Keywords:** rheumatoid arthritis, gut microbiota, gut–joint axis, microbiota-derived metabolites, mucosal immunity

## Abstract

**Highlights:**

**What are the main findings?**

- RA-associated gut dysbiosis is characterized by stage-dependent functional ecological remodeling rather than taxonomic shifts alone.
- Microbiota-derived metabolites connect gut dysbiosis with epithelial barrier dysfunction, innate/adaptive immune imbalance, and joint inflammation.

**What are the implications of the main findings?**

- This review places RA within a broader gut–host organ communication framework relevant to inflammatory diseases.
- Mechanism-informed microbiome and metabolite markers may support RA risk stratification, response prediction, and adjunctive intervention design.

**Abstract:**

The gut microbiota is a key regulatory hub linking environmental exposure, the mucosal barrier, and joint inflammation, and plays an important role in the pathogenesis and progression of rheumatoid arthritis (RA). Mechanistic studies in this field mainly address two interrelated questions: how RA-associated gut microbiota modulate mucosal immunity and systemic autoimmunity through strain-level variation, niche competition, and metabolic remodeling; and how disease stage, host immune status, and drug exposure reciprocally reshape gut microbial structure and function. Accordingly, this review follows the framework of “anti-inflammatory/pro-inflammatory microbial niches—microbiota-derived metabolites—immune cell homing and migration” to summarize recent advances in the role of gut microbiota and their derivatives in RA onset, progression, and therapeutic response. Focusing on disease-stage-specific remodeling of gut functional ecology, we discuss how short-chain fatty acids, tryptophan-derived indoles, bile acids, succinate, and other microbial effector molecules regulate RA immunopathology through regulatory T cells (Treg), regulatory B cells (Breg), type 17 T helper cells (Th17), and IL-17-producing T follicular helper cells (Tfh17), dendritic cells, fibroblast-like synoviocytes, and osteoclasts. We also highlight intestinal antigen sampling, autoantibody generation, immune cell trafficking, and synovial reactivation as key links in the gut–joint axis. This review aims to shift RA microbiome research from taxonomic profiling toward stage-specific functional ecological analysis, providing a basis for risk stratification, therapeutic response prediction, and microbiota-based adjunctive interventions.

## 1. Introduction

Rheumatoid arthritis (RA) is a chronic autoimmune disease characterized by synovitis, bone and cartilage destruction, systemic immune dysregulation, and long-term functional impairment [1]. In recent years, RA has increasingly been recognized as a disease with a continuous evolutionary trajectory rather than a single event beginning only at the onset of clinically apparent arthritis. Individuals who are positive for anti-cyclic citrullinated peptide antibodies (anti-CCP) and present with new-onset musculoskeletal symptoms but have no clinical synovitis have been used to define populations at high risk for RA [2]. In such populations, longitudinal cohort studies have shown that the gut microbiota of individuals who subsequently progress to clinical arthritis may undergo marked fluctuations within approximately 10 months before disease onset, characterized by increased Bray–Curtis dissimilarity and reduced microbiota stability. By contrast, microbiota composition remains relatively stable in non-progressors and in individuals at earlier time points before arthritis onset, suggesting that RA may have an identifiable and traceable preclinical microbial ecological window [3]. Meanwhile, an increasing body of evidence supports the hypothesis that RA-related autoimmunity may originate at mucosal sites, including barrier tissues such as the oral cavity, lung, and gut. Among these, the gut has emerged as a critical interface linking environmental factors to systemic autoimmunity because of its enormous microbial burden, abundant immune-cell networks, and continuous sensing of exogenous antigens and metabolites [4]. Therefore, incorporating the gut microbiota into studies of RA pathogenesis provides an important entry point for investigating where autoimmunity is initiated, how it is amplified, and why it influences drug responses. Existing evidence indicates that RA-associated alterations in the gut microbiota are strongly dependent on disease stage. In early untreated RA, the expansion of *Prevotella copri* and its immunological relevance first attracted considerable attention; subsequent studies showed that both the oral and gut microbiota are perturbed in patients with RA, and that some microbial abnormalities may partially normalize after treatment [5,6]. However, findings regarding *Prevotellaceae* or *Prevotella copri* have not been fully consistent across different cohorts. Cross-sectional and longitudinal studies in individuals at high risk for RA have shown that the association between Prevotellaceae abundance and RA progression is influenced by intrinsic risk factors, disease stage, and temporal proximity to disease onset, suggesting that Prevotellaceae may participate in RA-related microbial ecological alterations but does not dominate all microbiota changes [3]. Accordingly, disease stage should therefore frame analyses of microbial community structure, strain lineages, functional pathways, and metabolic outputs.

RA microbiome research is therefore shifting from taxonomic differences to functional ecology, including strain-level genomic features, metabolic capacity, niche competition, barrier interactions, and host immune coupling. Taking *Prevotella copri* as an example, subsequent studies have shown that the genomic lineages of different *Prevotella copri* strains or clades may determine their pathogenic potential, and that species-level abundance alone is insufficient to explain its relationship with RA [7]. In addition, multi-omics studies have revealed distinct microbiota–metabolic abnormalities at different stages of RA. For example, reductions in *Bacteroides uniformis* and *Bacteroides plebeius* are associated with impaired glycosaminoglycan metabolism; increased *Escherichia coli* is associated with enhanced arginine succinyltransferase pathway activity and rheumatoid factor levels; and persistently increased intestinal permeability may be related to the presence of microbial signals in the synovial fluid of patients with late-stage RA [8]. These findings suggest that the gut microbiota may jointly contribute to a functional ecological framework for RA onset and progression by influencing joint matrix metabolism, bone destruction, mucosal barrier integrity, and autoantibody generation. Within this functional ecological framework, microbiota-derived metabolites act as key mediators linking microbial networks to host immune effector responses. Short-chain fatty acids, tryptophan-derived indole metabolites, bile acids, succinate, and other microbial effector molecules may participate in RA progression by regulating barrier function, antigen presentation, T/B-cell differentiation, fibroblast-like synoviocyte activation, and bone metabolism [9]. Previous studies have shown that microbial-derived butyrate can suppress excessive immune activation in the intestinal mucosa, promote the differentiation and function of anti-inflammatory regulatory T cells, and inhibit arthritis by enhancing aryl hydrocarbon receptor (AhR) signaling in regulatory B cells [10]. More recent work further demonstrated that propionate can alleviate experimental arthritis by targeting the HDAC3–FOXK1–interferon axis in fibroblast-like synoviocytes, an effect supported by the upstream propionate-producing bacterium *Bacteroides* fragilis [11]. In addition, *Bifidobacterium* pseudocatenulatum can attenuate joint injury in the collagen-induced arthritis model by reshaping bile acid metabolism, enhancing G protein-coupled bile acid receptor 1, also known as TGR5 (GPBAR1/TGR5) signaling, and suppressing aberrant Th1/Th17 responses [12]. Thus, microbial metabolites are both outputs of gut functional ecology and effectors of systemic and joint inflammation.

Gut microbial effects in RA extend beyond increased intestinal permeability. Microbial antigens and metabolites can initiate mucosal immune responses that are subsequently propagated and amplified in the joint. Segmented filamentous bacteria, for example, induce Th17 or Peyer’s patch-derived Tfh responses and promote arthritis through immune-cell migration and systemic autoantibody production [13,14]. Meanwhile, *Fusobacterium nucleatum* can exacerbate RA-like inflammation through FadA-containing outer membrane vesicles; *Eggerthella lenta* can enhance preclinical autoantibody production and aggravate experimental arthritis; and clonal IgA/IgG autoantibodies derived from individuals at high risk for RA can recognize specific gut Subdoligranulum strains and induce arthritis-like phenotypes [15,16,17]. Together, antigen sampling, dendritic-cell-mediated T-cell differentiation, Th17/Tfh17–Treg/Tfr imbalance, B-cell class switching, and autoantibody expansion can convert intestinal signals into inflammation within a joint niche composed of fibroblast-like synoviocytes, macrophages, and osteoclasts. The gut microbiota may also influence therapeutic responses in RA. Studies in patients with new-onset RA have shown that pretreatment gut microbiota composition is associated with non-response to methotrexate, indicating the potential of the microbiota to predict initial therapeutic outcomes [18]. Another study demonstrated that methotrexate acts not only on the host immune system but also on conserved metabolic pathways in multiple human-derived gut bacteria. Moreover, transplantation of the microbiota from methotrexate-treated patients into germ-free mice reduced immune activation after inflammatory challenge, indicating bidirectional interactions among drugs, the microbiota, and host immunity [19]. Further studies in large RA cohorts suggest that pretreatment oral and gut microbiomes can predict disease-modifying antirheumatic drugs (DMARDs) responses, and that post-treatment microbial changes differ between responders and non-responders [20]. Therefore, intervention strategies targeting the gut microbiota and its metabolites should be understood within the framework of current treat-to-target RA management. Their rational role is not to replace DMARDs, but to support disease risk stratification, response prediction, therapeutic sensitization, and adjunctive management of difficult-to-treat RA.

In summary, this review follows the conceptual framework of “anti-inflammatory/pro-inflammatory microbial niches—microbiota-derived metabolites—immune cell homing and migration” to systematically summarize the roles of the gut microbiota and its derivatives in RA onset, progression, and therapeutic response. We first analyze stage-dependent functional ecological remodeling, then trace how microbial metabolites and mucosal immune responses reach and amplify within the joint, and finally assess microbiota-targeted adjunctive interventions. The aim is to move beyond taxonomic association toward identifying which microbial functions act at which disease stages, through which metabolites and immune pathways, and in which patient subgroups.

## 2. Functional Ecological Remodeling of the Gut Microbiota Across Different Disease Stages of RA

The RA-associated gut microbiota should not be regarded as a fixed disease label, but rather as a dynamic ecosystem that changes continuously during genetic susceptibility, preclinical autoimmunity, initial inflammation, chronic activity, and therapeutic intervention. Previous studies have often relied on 16S rRNA sequencing or species-level abundance differences to identify characteristic taxa that are increased or decreased in patients with RA compared with healthy controls. However, as study populations have expanded from new-onset untreated RA to preclinical high-risk individuals, active RA, long-term treated RA, and populations with diverse geographic, dietary, and drug-exposure backgrounds, directional changes in a single genus or species have not always been consistent. Therefore, the key question in RA gut microbiota research is shifting from which taxa are increased or decreased to which microbial communities exert immunological effects at specific disease stages, under particular host backgrounds, within defined ecological niches, and through specific combinations of functional genes. The substantial heterogeneity observed in RA microbiome studies may also reflect the fact that RA-associated dysbiosis is not a stable taxonomic signature, but a functional ecosystem jointly shaped by disease stage, strain lineage, metabolic pathways, mucosal barrier status, antigen-presentation context, and drug exposure.

### 2.1. Gut Microecological Alterations in Genetic Susceptibility and RA High-Risk Populations

RA susceptibility and high-risk populations mainly include first-degree relatives of patients with RA, HLA-DRB1 shared epitope carriers, and anti-CCP-positive individuals who have not yet developed clinical synovitis. Existing studies suggest that RA-associated gut microecological alterations may occur before the onset of clinical arthritis and are not entirely secondary to overt inflammation or pharmacological treatment. A cross-sectional study constructed a polygenic risk score based on 233 RA-associated genome-wide association study loci in RA-free participants from the Twins UK cohort and validated the findings in the SCREEN-RA first-degree relative cohort. The study found that *Prevotella* spp. were significantly positively associated with RA genetic risk scores; in SCREEN-RA, *Prevotella* spp. were also associated with HLA-DRB1 shared epitope risk alleles and the preclinical RA stage [21]. These findings suggest that RA-associated genotypes may establish links with specific microbial community structures before disease onset by shaping the mucosal immune milieu, antigen-presentation background, and gut ecological niches.

In anti-CCP-positive high-risk individuals with new-onset musculoskeletal symptoms but without clinical synovitis, the gut microbiota already exhibits features distinct from those of healthy controls. One study found significant differences at the microbial family level in high-risk individuals, characterized by enrichment of Lachnospiraceae, Helicobacteraceae, Ruminococcaceae, Erysipelotrichaceae, and Bifidobacteriaceae, while individuals who later progressed to RA formed clusters within phylogenetic networks [22]. These findings suggest that specific microbial community patterns may already exist months before RA onset, rather than abnormalities in a single species alone. A further longitudinal study including 124 anti-CCP-positive high-risk individuals with new-onset musculoskeletal symptoms but no clinical synovitis, of whom 30 progressed to RA, found that progressors exhibited gut microbiota instability approximately 10 months before RA onset, whereas non-progressors did not show similar changes [3]. Thus, preclinical RA may contain a traceable window of microbial ecological fluctuation, the core feature of which is not the persistent increase in a particular genus, but rather reduced microbial network stability and enhanced coupling with host immune-risk backgrounds.

Beyond the bacterial microbiome, the gut virome in RA high-risk populations has also begun to attract attention. Studies have shown that, among first-degree relatives at risk of RA, gut bacteriophage communities may diverge according to anti-CCP status and are enriched for phages associated with Streptococcaceae, Bacteroidaceae, and Lachnospiraceae. Auxiliary metabolic genes carried by these phages are associated with anti-CCP status, suggesting that the virome may participate in the preclinical stage of RA by modulating bacterial metabolic potential and immunoregulatory capacity [23]. Accordingly, gut microecological alterations in genetic susceptibility and RA high-risk states can be understood as a co-evolutionary process involving genetic risk, the mucosal immune environment, the bacterial microbiome/virome, and metabolic functions, rather than as a simple abnormality in bacterial abundance.

### 2.2. Microbial Features in New-Onset Untreated RA

After progression to new-onset untreated RA, patients have developed clinically recognizable synovitis and systemic inflammation but have not yet undergone substantial remodeling by DMARDs, biologics, or other therapies. This stage therefore represents the microbial ecological window most closely associated with the transition from preclinical autoimmunity to overt arthritis [20]. Metagenomic studies have shown that the fecal, dental plaque, and salivary microbiota of patients with RA deviate from those of healthy controls, suggesting concordant alterations in both gut and oral microbial ecosystems. Among these changes, *Haemophilus* spp. are reduced across multiple body sites in RA and are negatively correlated with autoantibody levels, whereas *Lactobacillus salivarius* is enriched at multiple sites in patients with RA, particularly among those with higher disease activity [6]. These findings indicate that new-onset RA is not characterized solely by abnormalities in a single gut ecological niche, but may involve coordinated dysbiosis across multiple mucosal sites, including the oral cavity and gut.

Signals related to *Prevotella copri* were among the earliest microbial features to attract attention in new-onset RA. Early studies linked the expansion of *Prevotella copri* to new-onset RA [5], but subsequent work suggested that species-level abundance alone is insufficient to explain its relationship with RA. An isolation and comparative genomics study showed that *Prevotella copri* strains derived from patients with RA harbor specific genomic insertion regions involving conjugative transposon-related sequences and exhibit stronger pro-arthritogenic capacity in animal models [7]. Further studies also demonstrated that RA-derived and health-derived *Prevotella copri* strains differ not merely in species identity, but also in their pangenome, mobile genetic elements, and immunostimulatory capacity, with RA-derived strains being more prone to induce stronger IL-6 and IL-23 responses and stronger inflammatory responses [7]. Therefore, the key issue in new-onset RA is not simply whether *Prevotella copri* is increased, but which *Prevotella copri* strains and functional genes are present and how they interact with the host immune background.

Strain specificity is also reflected at the level of antigenic immune recognition. Studies have shown that some patients with RA have specific IgG or IgA immune responses against *Prevotella copri*, a response that is less common in other rheumatic diseases and healthy controls [24]. Further work has identified HLA-DR-presented immunogenic peptides derived from *Prevotella copri*, suggesting that gut bacterial antigens may enter the RA autoimmune network through HLA-restricted antigen presentation [25]. In addition to *Prevotella copri*, clonal autoantibodies derived from IgA/IgG double-positive plasmablasts in RA high-risk individuals can recognize specific Subdoligranulum strains, which can activate RA-associated T cells and induce arthritis-like phenotypes in mice [17]. Together, these studies indicate that key microbial signals in new-onset and preclinical RA need to be understood at the levels of strain identity, antigenicity, and immune recognition.

Functional evidence further supports this paradigm shift. A cohort study of women with early RA found reduced gut microbial diversity, relative enrichment of Bacteroidetes, and higher Actinobacteria and *Collinsella* abundance in healthy controls. Functional prediction suggested enrichment of iron-transport-related genes in early RA and lipopolysaccharide biosynthesis-related genes in clinically overt RA [26]. This indicates that, as high-risk states progress to overt RA, the microbiota may shift from changes related to immune initiation toward enhanced functional states involving endotoxin burden, metal ion transport, redox metabolism, and inflammatory metabolism. Another multi-omics study proposed that, in early RA, the gut microbiota may contribute to disease progression by enhancing ascorbate degradation [27], suggesting that microbial abnormalities in new-onset untreated RA may promote synovial inflammation and osteochondral damage by weakening antioxidant capacity, collagen synthesis, and matrix homeostasis. In addition, expansion of low-abundance or rare-lineage microbes can also be observed in new-onset RA. For example, *Eggerthella lenta* is associated with RA severity, antibody responses, and metabolically aged inflammation, and can enhance rheumatoid factor and collagen-related antibody responses in humanized mouse models, accompanied by alterations in bile acid, amino acid, and nucleotide metabolism [16]. Therefore, gut microecological changes in new-onset untreated RA can be summarized as the expansion of *Prevotella*-related communities and specific pathogenic strains, reduction in some beneficial commensals and mucosal-homeostasis-associated bacteria, multi-site oral–gut dysbiosis, expansion of low-abundance immunologically active microbes, and remodeling of functional pathways related to lipopolysaccharide, iron transport, ascorbate degradation, and bile acid metabolism.

### 2.3. Microbial Remodeling in Established Active, Chronic, and Post-Treatment RA

When patients enter established active RA, chronic RA, or post-treatment states, the gut microecology is no longer only a potential trigger before disease onset, but gradually becomes a dynamic system mutually shaped by persistent inflammation, barrier injury, drug exposure, disease burden, and therapeutic response. A nested case–control study of 40 patients with RA and 40 sex- and age-matched controls found a trend toward separation in β-diversity between patients with RA and controls. The RA group showed increased *Enterococcus*, *Sedimentibacter*, and *Collinsella*, reduced *Dorea formicigenerans*, and increased predicted genes related to arginine deiminase, a substantial proportion of which were attributed to *Collinsella*. Further analysis showed that *Collinsella aerofaciens* was independently associated with RA-related factors such as high anti-citrullinated protein antibodies (ACPA), smoking, and age [28]. These findings suggest that microbial changes in active or established RA may contribute to maintaining an immune background characterized by local intestinal citrullination, autoantigen exposure, and ACPA responses, rather than merely accompanying inflammation passively.

In a study of 110 patients with RA and 110 matched controls, investigators further stratified patients according to disease activity and found differences in microbial composition and predicted metabolic pathways among the moderate/high disease activity group, remission/low disease activity group, and controls. Multivariable analysis indicated that factors associated with cumulative inflammatory activity included age, obesity, HAQ functional score, and *Collinsella* expansion [29]. Therefore, the association between *Collinsella* expansion and inflammatory status may represent a noteworthy clue in active RA, particularly under conditions of long-term inflammatory exposure. For chronic RA, a study including 94 patients with established RA treated for at least 1 year and 30 healthy controls found that the overall microbial composition of patients with RA differed from that of healthy individuals. However, after stratification by Disease Activity Score in 28 joints (DAS28), no significant differences in α- or β-diversity were observed among remission, low, moderate, and high disease activity RA groups, and ACPA or RF status had limited effects on overall microbial structure [30]. This suggests that, in established RA, differences between having RA and not having RA may be more stable than differences between gut microbiota composition and current DAS28 levels.

IL-23 may provide an immune link between microbial remodeling and inflammatory activity in RA. Circulating IL-23 levels are elevated in early RA and correlate with disease activity [31,32], although direct paired human evidence linking IL-23 concentrations to individual gut taxa remains limited. Mechanistically, RA-derived *Prevotella copri* strains induce greater IL-6 and IL-23 production in dendritic cells and more severe arthritis than strains isolated from healthy controls [7]. These observations support a microbiota–IL-23/Th17 axis while underscoring the need for longitudinal studies integrating microbial composition, circulating or mucosal IL-23 levels, and disease activity.

Compared with simple genus-level abundance, a more explanatory mechanism in chronic RA may involve a persistent state of chronic inflammation caused by ongoing impairment of gut barrier function and trans-barrier exposure to microbial products. Studies have shown that circulating bacterial DNA and intestinal fatty acid-binding protein are elevated in the blood of patients with RA, suggesting increased bacterial translocation and intestinal epithelial injury, while fecal proteomic analysis reveals increased host inflammatory proteins [33]. Thus, gut microecological abnormalities in chronic RA should not be understood merely as increases or decreases in certain taxa, but rather as a pathological ecological niche formed by barrier disruption, translocation of microbial components, release of host inflammatory proteins, and sustained systemic immune activation.

Post-treatment microbial remodeling in RA mainly involves two directions. First, anti-inflammatory therapy may indirectly promote microbiota recovery by reducing TNF-α-, IL-6-, and IL-17-related inflammation and gut barrier injury. Second, baseline microbial structure may influence drug metabolism, immune responses, and treatment outcomes, thereby serving as a predictor of therapeutic efficacy. In established RA, methotrexate (MTX), leflunomide, and hydroxychloroquine have no significant effect on overall microbial composition, whereas sulfasalazine can reduce microbial richness and evenness, increase *Lactobacillus sakei*, the Christensenellaceae R7 group, and *Coprococcus*, and reduce *Clostridium*, *Roseburia*, and *Turicibacter* [30]. Because sulfasalazine requires activation by bacterial azoreductases, these findings suggest a clear drug–microbe interaction for certain DMARDs. Meanwhile, patients treated with TNF inhibitors show higher abundance of potential short-chain fatty acids (SCFAs)-producing bacteria, including *Anaerostipes*, *Subdoligranulum*, and the Lachnospiraceae ND3007 group, compared with those treated with abatacept or tocilizumab [30], suggesting that microbiota changes after TNF blockade may favor restoration of SCFA-related ecological functions. Another study showed that, compared with untreated RA patients, the etanercept-treated group exhibited partial restoration of beneficial microbiota, including increased Cyanobacteria/Nostocales and decreased Deltaproteobacteria and Clostridiaceae; the MTX group mainly showed decreased Enterobacteriales, indicating that anti-TNF therapy may partially correct RA-associated microbial abnormalities [34]. In addition, among patients with moderate to high disease activity followed for 6 months after treatment adjustment, good responders to DMARDs had higher baseline *Fusicatenibacter*, *Subdoligranulum*, and Clostridia UCG-014, whereas higher *Faecalitalea* was associated with poor response. The combination of *Fusicatenibacter* and *Subdoligranulum* predicted DMARDs response with an area under the curve (AUC) of 0.807 [30]. These findings suggest that the gut microbiota is not only a concomitant indicator of disease status, but may also become an auxiliary stratification tool for deciding whether to continue intensifying csDMARD therapy or switch to biologics.

### 2.4. Strains, Niches, and Metabolic Pathways Shape the Functional Ecology Underlying Stage-Specific Differences

The marked heterogeneity of microbial changes across different disease stages arises fundamentally because the RA gut microbiota is not determined by a single organism, but by a functional ecosystem composed of strain specificity, nutrient substrates, redox state, mucus layer, bile acid pool, SCFA levels, barrier integrity, and mucosal immune tone. In other words, the same microorganism may produce different consequences in different host environments, and the same nutrient substrate may be directed toward distinct metabolic products and immune effects in different microbial ecosystems. Therefore, RA gut microbiota research needs to move beyond taxonomic differences toward comparisons between pro-inflammatory and protective ecological niches.

Experimental evidence for pro-inflammatory niches first came from animal models. In the K/BxN arthritis model, arthritis is markedly attenuated under germ-free conditions, but colonization with segmented filamentous bacteria can restore or exacerbate arthritis by inducing Th17 cells, driving autoimmune inflammation at distant joint sites [13]. Clinical data also support this view. For example, Fusobacterium nucleatum is enriched in patients with RA and is associated with disease severity; its FadA-containing outer membrane vesicles can translocate to the joints, act on synovial macrophages, and activate the Rab5a–YB-1 inflammatory axis, thereby exacerbating collagen-induced arthritis [15]. However, pro-inflammatory niches are context-dependent and plastic. The conventional view holds that a high-fiber diet may help restore the gut microbiota of patients with RA toward a healthier state [35]. Yet, in the specific context of colonization by RA-derived *Prevotella copri*, a high-fiber diet can instead aggravate arthritis by altering microbial composition and enhancing intestinal inflammation [36]. This phenomenon suggests that substrates such as dietary fiber do not inherently exert unidirectional anti-inflammatory effects; their ultimate effects depend on the pre-existing microbiota, strain characteristics, metabolic pathways, and host immune background.

By contrast, the core function of protective niches lies in maintaining mucosal homeostasis and immune tolerance. Studies have shown that human-derived Prevotella histicola can suppress inflammatory arthritis in susceptible mouse models such as HLA-DQ8 mice. It promotes gut homeostasis, increases butyrate-producing bacteria such as Allobaculum, and improves SCFA-related metabolism, thereby reducing inflammation and improving motor function [37]. This forms an important contrast with *Prevotella copri*, indicating that different species or strains within the same genus Prevotella may occupy either pro-inflammatory or protective ecological niches. Studies on Bacteroidales have shown that specific members of this order can recruit IL-6-producing intestinal intraepithelial lymphocytes and promote colonic barrier integrity, suggesting that IL-6 is not always pro-inflammatory in the gut microenvironment, but may also participate in tissue repair and barrier maintenance [38]. SCFAs, particularly butyrate, provide a key functional clue for protective ecological niches. Reduced microbiota-derived SCFAs have been observed in both patients with RA and arthritic mice [37], and butyrate supplementation can alleviate arthritis symptoms in both clinical patients and animal models [39,40]. SCFA-producing bacteria and their interactions with cross-feeding microbes form an important basis for maintaining protective gut ecological niches [41]. In addition, protective niches may also exhibit temporal rhythmicity. Studies have shown that feeding time can induce circadian oscillations in the gut microbiota and regulate inflammatory rhythms in RA; among these changes, *Parabacteroides* distasonis can respond to nutritional cues by releasing glycitein and attenuate inflammation through the SIRT5–NF-κB axis [42]. This suggests that protective ecological niches are not only defined by the spatial presence of specific microbes, but also by the alignment between microbial metabolic activity and host circadian immune rhythms.

What truly determines pro-inflammatory or protective effects is often not the taxonomic name itself, but the functional genes and metabolic pathways carried and actually expressed by the microbial niche, as well as their coupling with host immune receptors. Metagenomic studies have shown that patients with RA and healthy controls may not always differ significantly in α- or β-diversity, yet disease-associated alterations may still be evident at the level of gene function and metabolic pathways. For example, a metagenome-wide association study in an RA population found that, in addition to higher abundance of genera such as Prevotella in RA, the redox-related gene R6FCZ7 was significantly reduced in the RA metagenome. The study also observed enrichment of metabolic pathways such as fatty acid biosynthesis and glycosaminoglycan degradation in case–control comparisons, although no obvious differences in overall α- or β-diversity were detected between RA and control groups [43]. Another metagenomic study showed that reductions in *Bacteroides uniformis* and *Bacteroides plebeius* were associated with weakened glycosaminoglycan metabolism, which may continuously affect cartilage matrix homeostasis, whereas increased E. coli was associated with enhanced arginine succinyltransferase pathway activity and correlated with elevated rheumatoid factor (RF) and bone loss [8]. These findings further indicate that specific microbial functions may exert sustained effects on the onset and progression of RA.

Overall, RA-associated functional pathways can be summarized into five categories. The first comprises carbohydrate-active enzymes, complex polysaccharide utilization loci, and SCFA-generating pathways, which determine whether dietary fiber, host mucin glycans, and glycosaminoglycans can be appropriately degraded and converted into immunoregulatory metabolites such as acetate, propionate, and butyrate [44]. If protective polysaccharide fermentation networks are weakened, epithelial energy supply, mucus layer homeostasis, and Treg/Breg induction capacity may all decline. In addition, higher serum SCFA levels in RA high-risk individuals are associated with a lower risk of clinical progression, suggesting that this pathway may participate in RA risk modulation [45]. The second category involves mucus and host glycan degradation pathways. Moderate utilization of host glycans can maintain a commensal ecosystem; however, under conditions of dietary fiber deficiency or inflammatory stress, excessive mucus degradation may weaken the barrier and increase exposure to microbial products. Once intestinal permeability is increased, microbial components such as LPS, peptidoglycan, and outer membrane vesicles can more readily cross the barrier and activate local immune cells [4]. The third category involves redox- and iron-transport-related pathways. Inflammatory environments are often accompanied by increased nitrate, oxygen, and reactive oxygen species, which favor the expansion of facultative anaerobes and inflammation-adapted bacteria, thereby forming pro-inflammatory ecological niches that promote the onset and progression of RA [46,47]. The fourth category involves arginine, citrulline, and nitrogen metabolism pathways. One of the central immunological events in RA is the generation of citrullinated antigens and ACPA responses; microbial arginine metabolism, arginine deiminase-like activity, or related metabolic bypasses may alter local antigen modification and the autoantibody background [48]. The fifth category includes pathways involved in LPS, peptidoglycan, outer membrane vesicles, and bacterial lipid synthesis. These molecules can function as innate immune stimuli, activate TLRs, NOD-like receptors, macrophages, and dendritic cells, promote Th17/Tfh and B-cell responses, enhance systemic autoantibody responses, and aggravate RA [45].

**Figure 1:**
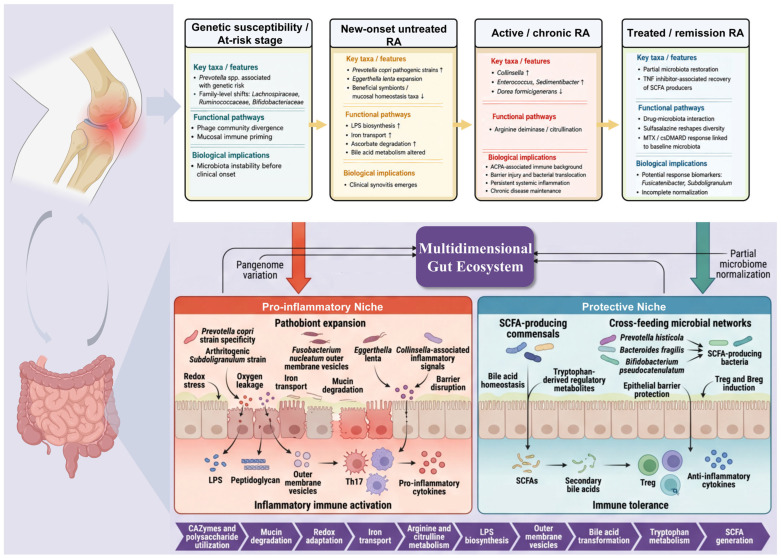
Disease-stage-dependent transition from taxonomic dysbiosis to functional microbial ecology in rheumatoid arthritis. Disease stage, inflammation, barrier injury, and antirheumatic treatment reshape microbial ecology, whereas microbial functions and metabolites may reciprocally influence autoantibody generation, mucosal immune activation, synovial inflammation, and therapeutic response. ↑ indicates an increase in abundance; ↓ indicates a decrease in abundance.

In summary, disease stage determines the timing and clinical meaning of microbial abnormalities, whereas functional ecology explains why taxa or substrates can produce different outcomes across studies (Figure 1). RA microbiome research should therefore prioritize stage-specific strain–niche–pathway–metabolite relationships over taxonomic abundance alone.

## 3. Microbiota-Derived Metabolites Mediate the Link Between Functional Ecology and Host Immunity

**Figure 2:**
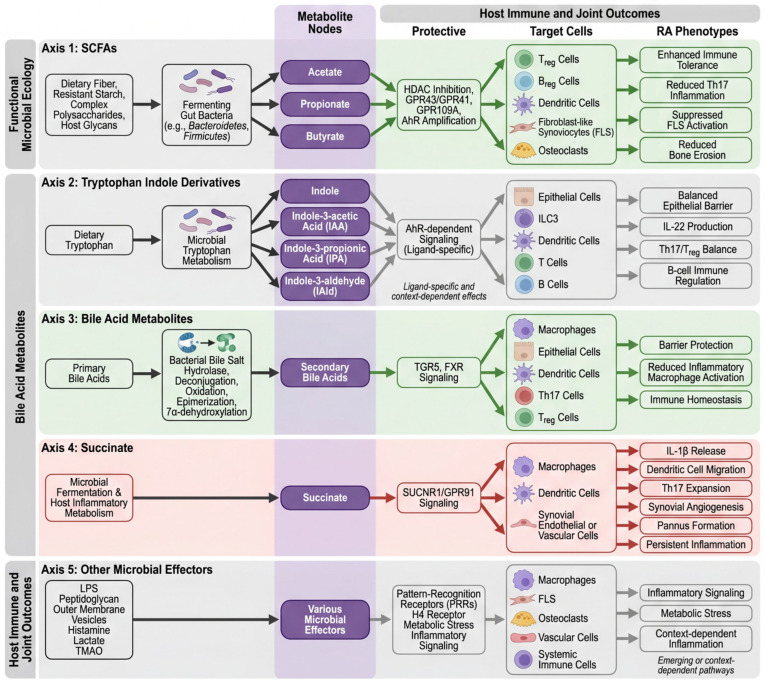
Microbiota-derived metabolites as mechanistic and translational links between gut functional ecology and rheumatoid arthritis immunopathology.

Microbiota-derived metabolites occupy a pivotal position in linking upstream microbial ecology with downstream host immune responses. On the one hand, they represent functional outputs generated from dietary fiber, bile acids, tryptophan, amino acids, host mucus glycans, and other substrates through microbial enzymatic processing. On the other hand, they can act on intestinal epithelial cells, dendritic cells, T/B cells, macrophages, fibroblast-like synoviocytes, and osteoclasts through G protein-coupled receptors, nuclear receptors, transcription factors, and epigenetic regulatory mechanisms, ultimately influencing immune tolerance, synovial inflammation, and bone destruction. The following section discusses the evidence and mechanisms of SCFAs, tryptophan-derived indole metabolites, bile acids, succinate, and other metabolites in RA pathogenesis and treatment. Please see Figure 2 for the illustration.

### 3.1. SCFAs Regulate Breg Cells, Treg Cells, and Fibroblast-like Synoviocytes

SCFAs are mainly produced by intestinal anaerobes through fermentation of dietary fiber, resistant starch, and certain host-derived glycans, with acetate, propionate, and butyrate as representative members. Their upstream ecological basis consists of a fermentation network involving complex polysaccharide-degrading bacteria, lactate/succinate cross-feeding bacteria, and butyrate- and propionate-producing communities [44]. Studies have shown that butyrate promotes colonic peripheral Treg differentiation and supports immune tolerance by enhancing histone acetylation at the Foxp3 locus [49]. Propionate can also enhance peripheral Treg generation [50], indicating that SCFAs are important mediators through which the microbiota regulates mucosal immune homeostasis. Further studies have demonstrated that microbiota-derived SCFAs, particularly butyrate, are reduced in patients with RA and arthritic mice. Butyrate supplementation alleviates arthritis in mice, and this protective effect depends on Breg cells and is associated with butyrate-induced increases in 5-hydroxyindole-3-acetic acid, which further activates AhR signaling in Breg cells [10]. Given the frequent presence of impaired Treg function and excessive Th17 activation in RA, studies have shown that oral butyrate markedly attenuates collagen-induced arthritis in mice. This therapeutic effect is highly dependent on the Treg/IL-10/Th17 regulatory axis, and butyrate can directly promote the differentiation of naïve CD4^+^ T cells into Treg cells [51]. In addition, one study analyzed gut metagenomic data from twins with identical genetic backgrounds. Because RA concordance in twins is only 12–15%, gut metabolites may act as important non-genetic contributors to disease development. In two cohorts, unaffected twins were enriched for multiple SCFA-producing bacteria and exhibited more active SCFA metabolic pathways. Compared with their affected twin siblings, unaffected individuals had significantly higher fecal levels of butyrate, propionate, and acetate [52]. These findings suggest that fecal SCFAs and SCFA-producing gut bacteria may serve as key biomarkers distinguishing healthy individuals from patients with RA. With regard to propionate specifically, studies have shown that *Bacteroides fragilis* and propionate levels are higher in healthy individuals and collagen-induced arthritis (CIA)-resistant mice, and that B. fragilis or propionate can alleviate CIA. Mechanistically, propionate acts on RA-fibroblast-like synoviocytes (FLS), interferes with the HDAC3–FOXK1 interaction, promotes FOXK1 acetylation and degradation, and suppresses interferon signaling and tumor-like activation of FLS [11]. These findings indicate that SCFAs not only regulate systemic immunity through Treg/Breg pathways but can also directly reshape the inflammatory phenotype of synovial stromal cells. In therapeutic studies, oral butyrate, propionate, and acetate have all shown therapeutic effects in CIA animal models [53].

In summary, the SCFA–Breg/Treg/FLS axis can be conceptualized as follows: dietary fiber and complex polysaccharide substrates are converted by protective fermentative microbial communities into SCFAs such as butyrate and propionate. SCFAs promote Treg cells through HDAC inhibition and Foxp3 epigenetic regulation, enhance Breg function through the 5-hydroxyindole-3-acetic acid (5-HIAA)–AhR pathway, and directly inhibit RA-FLS and osteoclast activation, ultimately reducing autoantibody responses, synovial inflammation, and bone destruction.

### 3.2. Tryptophan-Derived Indole Metabolites Regulate Mucosal Immunity Through AhR

Tryptophan is another key substrate connecting diet, the microbiota, and immune responses. Host cells can convert tryptophan into kynurenine and its derivatives through the indoleamine 2,3-dioxygenase 1 (IDO1)/tryptophan 2,3-dioxygenase (TDO) pathway, whereas the gut microbiota can generate indole, indole-3-propionic acid, indole-3-acetic acid, indole-3-aldehyde, indole-3-pyruvic acid, and other metabolites through tryptophanase, aromatic amino acid aminotransferases, and related pathways. Many indole derivatives act as AhR ligands and can be sensed by intestinal epithelial cells, ILC3s, dendritic cells, T cells, and B cells, thereby regulating barrier integrity, IL-22 production, Treg/Th17 balance, and B-cell immune responses [54]. Studies have shown that indole-3-pyruvic acid exerts protective effects in both plasma metabolomic analyses of patients with RA and CIA animal models. Administration of indole-3-pyruvic acid alleviates CIA, and in vitro experiments show that it suppresses Th17 differentiation while promoting Treg differentiation; these effects can be attenuated by the AhR antagonist CH223191 [55], suggesting ligand-specificity within the indole metabolite–AhR axis. A therapeutic study further found that sinomenine can regulate the gut microbiota, particularly by increasing *Lactobacillus*-related bacteria, upregulate microbial tryptophan metabolites such as IA, IPA, and IAA, activate AhR, and restore Th17/Treg balance, thereby exerting microbiota-dependent anti-arthritic effects in the CIA model [56]. This indicates that this axis is also context-dependent. However, indole metabolites should not be simply interpreted as protective factors merely because they activate AhR. In the CIA model, microbiota-dependent indole production was found to be associated with arthritis development. Under a low-tryptophan diet, indole supplementation increased serum IL-6, TNF, and IL-1β levels, increased RORγt^+^CD4^+^ T cells, enhanced IL-17 production after collagen stimulation, and promoted glycosylation changes in complement-fixing anti-collagen antibodies. IL-23 neutralization reduced indole-induced disease severity [57]. These findings suggest that indole precursors may promote Th17 and autoantibody responses under specific inflammatory conditions, contrasting with the protective effects of some indole derivatives. In addition, studies have shown that IAA is reduced in CIA rats, and that IAA supplementation can reduce Foxp3 ubiquitination and degradation through the AhR–TAZ–Tip60 pathway, promote Treg differentiation, and alleviate inflammation [58]. Together with the protective effects of IPA and IA/IPA/IAA mixed metabolites, these findings suggest that specific indole derivatives may exert anti-inflammatory effects by stabilizing Treg function and barrier immunity; however, indole itself may promote Th17- and antibody-mediated inflammation in CIA.

In summary, tryptophan-derived indole metabolites mainly influence barrier immunity, Treg/Th17 balance, and B-cell responses through AhR, but they show clear ligand specificity. Therefore, indole metabolites should not be regarded as unidirectional factors. Instead, specific metabolites such as indole, IPA, IAA, and IAld should be distinguished according to their receptor affinity, local concentration, and target-cell effects.

### 3.3. Bile Acids Maintain Barrier Immune Homeostasis Through TGR5 and FXR

Bile acids have both host-derived and microbiota-modified properties. The liver synthesizes primary bile acids from cholesterol and secretes them into the intestinal lumen after conjugation with glycine or taurine. The gut microbiota then converts them into unconjugated and secondary bile acids through bile salt hydrolase, 7α-dehydroxylation, oxidation–reduction, and epimerization reactions. Bile acids not only participate in lipid absorption but also act as signaling molecules through receptors such as FXR, TGR5/GPBAR1, and VDR, thereby regulating the intestinal barrier, inflammasome activity, macrophage polarization, and Treg/Th17 balance [59]. Direct evidence for the bile acid axis in RA has mainly focused on TGR5. Studies have shown that TGR5 activation reduces inflammatory responses in peripheral blood mononuclear cells from patients with RA and alleviates inflammation in collagen II-induced arthritis mice [60]. Further work linking the bile acid–TGR5 axis to the gut microbiota found that Bifidobacterium pseudocatenulatum can regulate bile acid metabolism, enhance BSH-related functions, increase unconjugated and secondary bile acids, and activate TGR5 signaling, thereby protecting the intestinal barrier, suppressing aberrant CD4^+^ T-cell responses, and reducing joint damage [12]. These findings suggest that bile acids are not merely digestive molecules involved in the enterohepatic circulation, but metabolic signals that can be reshaped by specific microbial communities and transmitted to joint immunopathological processes. In addition, the regulation of T-cell differentiation by bile acids provides important mechanistic insights for RA. Studies have shown that specific bile acid metabolites can regulate Th17 and Treg differentiation [61], and further work has demonstrated that microbial bile acid metabolites can also regulate RORγ^+^ Treg homeostasis [62]. Although these studies were not all conducted in RA contexts, their mechanisms targeting Th17/Treg balance are highly relevant to RA immunopathology and provide useful clues for further investigation. Regarding the bile acid–FXR axis, multi-omics studies have shown that in *Bacteroides*-dominant RA subgroups, bacteria associated with BSH and 7α-HSDH are increased, bile acid profiles are abnormal, FXR signaling is impaired, and these alterations are associated with ACPA and bone erosion [63].

In summary, bile acids reshape FXR/TGR5 signaling through microbial BSH activity and secondary bile acid metabolism, thereby influencing barrier function, macrophages, and T-cell immunity. Future studies should further distinguish the specific effects of different bile acid molecules, such as CA, DCA, LCA, UDCA, and isoalloLCA, across different RA stages and immune-cell subsets.

### 3.4. Succinate Drives Inflammatory Metabolism and Synovial Responses Through SUCNR1

Succinate is an intermediate of the tricarboxylic acid cycle and can also be produced by the gut microbiota during anaerobic fermentation. Unlike SCFAs and bile acids, an important feature of succinate signaling in RA is that its source is not restricted to the microbiota. Inflammation-activated macrophages, hypoxic synovial tissue, and metabolically reprogrammed immune and stromal cells can all lead to succinate accumulation. Thus, succinate can be understood as a danger-associated signaling molecule linked to both microbial metabolism and host inflammatory metabolism [64]. succinate receptor 1 (SUCNR1/GPR91) is the canonical membrane receptor for succinate. Studies have shown that succinate is abundant in the synovial fluid of patients with RA. Extracellular succinate released by inflammatory macrophages can be sensed by GPR91, inducing macrophage IL-1β release and forming a pro-inflammatory feed-forward loop. GPR91-deficient mice exhibit reduced macrophage activation and lower inflammatory levels in antigen-induced arthritis [65]. Further studies have demonstrated that SUCNR1 deficiency alleviates immune-mediated arthritis by reducing dendritic-cell migration and lymph node Th17-cell expansion [66], indicating that the succinate–SUCNR1 axis acts not only on innate immune cells but also influences adaptive immunity through antigen presentation and Th17 expansion. At the local joint level, succinate has been shown to induce angiogenesis in RA synovium through metabolic reprogramming and the HIF-1α/VEGF axis [67], and angiogenesis is a key basis for pannus formation, sustained inflammatory-cell infiltration, and osteochondral destruction in RA.

In summary, the succinate–SUCNR1/GPR91 axis can be conceptualized as follows: under inflammatory and hypoxic conditions, succinate accumulates and activates macrophages, dendritic cells, and synovial vascular-associated cells through SUCNR1, thereby promoting IL-1β release, Th17 expansion, angiogenesis, and persistent synovial inflammation. Compared with SCFAs, this axis is more strongly associated with inflammation and tissue destruction. However, the extent of its microbial origin and its relative contribution to RA still require validation through isotope tracing, germ-free/colonization models, and integrated human metabolomic studies.

### 3.5. Other Microbiota-Derived Effector Molecules and RA-Related Evidence

In addition to the four core metabolite categories discussed above, the RA-associated gut microbiota may influence disease phenotypes through a variety of low-abundance or context-dependent metabolites. The first is histamine. Studies have reported that histamine and histamine H4 receptor (H4R) promote osteoclastogenesis in RA [68], although the contribution of gut microbial sources in RA remains to be verified. Second, microbial metabolite trimethylamine N-oxide has been proposed to link vascular dysfunction with RA [69], but this axis is more closely related to vascular complications of RA and should not be directly equated with a core pathogenic metabolite in RA. Third, microbial-derived structural molecules such as LPS, peptidoglycan, and outer membrane vesicles originate from Gram-negative bacterial outer membranes, bacterial cell walls, and bacterial vesicles. Strictly speaking, they are not classical small-molecule metabolites. However, they are closely associated with bacterial translocation, gut barrier damage, and microbial product exposure in chronic RA, and constitute an important mechanism linking the gut to systemic inflammation [45]. They can therefore be included as non-classical microbiota-derived effector molecules. Finally, lactate is of particular interest because it can be derived from microbial fermentation as well as from enhanced glycolysis in RA synovial cells and immune cells. In the hypoxic synovial microenvironment, lactate may be linked to HIF-1α, VEGF, and inflammatory amplification [70], and its sustained accumulation appears to be associated with increased disease burden in RA.

In summary, compared with SCFAs, indole metabolites, and bile acids, these metabolites in RA remain mostly at the stage of association or cross-disease extrapolation and still lack complete causal chains connecting specific microbes or enzymatic systems, metabolites, receptors, target cells, and RA phenotypes. Overall, microbiota-derived metabolites not only explain the necessity of moving RA microbiome research from taxonomic differences toward functional ecology, but also provide a mechanistic foundation for subsequent discussions of immune-cell trafficking, local joint inflammation, and precision intervention.

## 4. Propagation and Amplification of Gut Immune Signals into Joint Inflammation

The gut microbiota and its derivatives do not necessarily need to enter the joints directly to exert pathogenic effects. More commonly, microbial communities, metabolites, and microbe-associated molecular patterns first act on the intestinal mucosal immune system, triggering antigen sampling, antigen presentation, T/B-cell differentiation, antibody class switching, and effector-cell repositioning. Subsequently, preactivated immune cells, autoantibodies, inflammatory mediators, and some microbiota-derived signals enter the circulation and, under the combined influence of genetic susceptibility, the synovial microenvironment, mechanical stress, and local inflammatory cues, are amplified into persistent synovitis, bone erosion, and cartilage destruction.

### 4.1. Intestinal Antigen Sampling and DC-Mediated Initiation of Mucosal Immunity

The gut is one of the largest and most complex interfaces of antigen exposure in the body. Dietary antigens, commensal bacteria, opportunistic pathogens, microbial metabolites, and microbe-associated molecular patterns continuously interact with the intestinal epithelium and mucosal immune system, requiring intestinal immunity to maintain a dynamic balance between immune tolerance and inflammatory defense [71]. Peyer’s patches, isolated lymphoid follicles, mesenteric lymph nodes, and the lamina propria together constitute the intestinal network for antigen sampling and immune induction. M cells can transcytose luminal particulate antigens and microbial components across the epithelium into mucosa-associated lymphoid tissues. Lamina propria DCs can also capture luminal or epithelial-derived antigens and deliver them to mesenteric lymph nodes, thereby initiating subsequent T-cell differentiation and homing programs. Regional specialization of the intestinal immune system, antigen-sampling routes, and the involvement of M cells and DCs in antigen delivery within small-intestinal lymphoid tissues provide an important basis for mucosal immunity to influence distal autoimmunity [71,72].

Under homeostatic conditions, intestinal CD103^+^ DCs have strong tolerogenic capacity. These DCs promote Foxp3^+^ Treg differentiation under the combined influence of TGF-β, retinoic acid, and local epithelial-derived factors, and confer gut-homing properties such as α4β7 and CCR9 expression on T cells, thereby maintaining immune tolerance to food antigens and commensal bacteria. However, when the intestinal barrier is impaired, trans-barrier exposure to microbial products increases, TLR/NOD-like receptors are persistently activated, or pro-inflammatory factors such as IL-6, IL-1β, and IL-23 predominate, the antigen-presentation milieu of DCs can shift from a tolerogenic to an inflammatory state, thereby driving the expansion of Th17, Tfh17, and other pro-inflammatory helper T cells [73].

Therefore, intestinal antigen sampling and DC-mediated T-cell fate determination represent key steps through which microbial signals enter the adaptive immune system. Under homeostatic conditions, the CD103^+^ DC–retinoic acid/TGF-β–Treg axis helps maintain mucosal tolerance. In contrast, under dysbiosis, barrier injury, and persistent PRR stimulation, IRF4^+^CD103^+^CD11b^+^ DCs, IL-6/IL-23 signaling, and antigen-specific Th17 responses may drive mucosal immunity from a tolerant state toward an inflammatory state. This transition lays the foundation for the subsequent development of systemic autoimmune responses.

### 4.2. Imbalance Among Th17, Tfh17, Treg, and Tfr Cells Shapes Systemic Autoimmunity

After mucosal immune activation in the gut, the balance among Th17, Tfh17, Treg, and Tfr cells determines whether the local immune response returns to tolerance or progresses toward systemic autoimmunity. In the healthy state, the gut microbiota and its metabolites maintain the dynamic equilibrium among Th17, Tfh/Tfh17, Treg, and Tfr cells by preserving barrier integrity, regulating DC and mononuclear phagocyte function, and providing immunoregulatory signals such as SCFAs and tryptophan metabolites. Conversely, in an RA-susceptible background, dysbiosis, altered metabolite profiles, and increased barrier permeability can cause intestinal antigens and microbial products to persistently stimulate the mucosal immune system, enhance inflammatory signaling mediated by IL-6, IL-1β, IL-23, and TNF-α, and thereby promote the expansion of Th17 and Tfh17-like responses while weakening Treg/Tfr-mediated immune tolerance [45,74,75].

This immune deviation may precede the onset of clinically apparent arthritis. Studies have shown that individuals at high risk for RA already exhibit gut microbiota and serum metabolomic alterations before the development of clinical arthritis, and that FMT from preclinical individuals can induce intestinal barrier abnormalities and mucosal immune imbalance [76]. These findings suggest that gut immune dysregulation may not simply be secondary to RA inflammation, but may contribute to the autoimmune priming phase of preclinical RA. Th17 cells are central to this immune shift. In the K/BxN autoimmune arthritis model, germ-free conditions markedly attenuate arthritis, whereas colonization with segmented filamentous bacteria restores or exacerbates arthritis by inducing Th17 cells [13]. At the joint level, Th17 cells and their related cytokines amplify RA inflammation through multiple mechanisms. On the one hand, IL-17 can stimulate FLS, macrophages, and endothelial cells to release IL-6, TNF-α, CXCL1, CXCL8, CCL20, and matrix metalloproteinases. On the other hand, Th17-related signals can promote RANKL expression and osteoclastogenesis, thereby linking synovial inflammation with bone erosion [77,78,79].

In addition to Th17 cells, imbalance between Tfh17 and Tfr cells is another important mechanism linking the gut microbiota to systemic autoimmunity. The gut microbiota can promote the differentiation and migration of Peyer’s patch Tfh cells, enhance systemic autoantibody responses, and ultimately aggravate autoimmune arthritis [14]. By contrast, Tfr cells are essentially follicular Treg cells that restrict Tfh-driven abnormal germinal center responses and autoantibody expansion. Reduced Tfr cells have been observed in patients with new-onset RA, accompanied by gut dysbiosis and changes in microbiota-associated metabolites; among these, *Ruminococcus* and arachidonic acid have been proposed to be associated with RA status and disruption of Tfr-mediated immune tolerance [80]. This finding is important because Tfr insufficiency may allow gut-induced Tfh/Tfh17-like responses to more readily convert into systemic B-cell activation and the generation of autoantibodies such as ACPA and RF.

From the perspective of microbial metabolites, SCFAs are representative molecules linking gut ecology with the Th17/Treg balance. In the CIA model, butyrate intervention alleviates arthritis through the Treg/IL-10/Th17 axis. *Lactobacillus casei* CCFM1074 alleviates arthritis in CIA rats by regulating the gut microbiota, metabolites, and Treg/Th17 balance. Studies focusing on the FFA2 receptor have also shown that SCFAs can regulate B-cell differentiation and attenuate RA-like inflammation [51,53,81]. These findings indicate that the imbalance among Th17, Tfh17, Treg, and Tfr cells is not a fixed immune state, but a dynamic process that can be reshaped by microbial composition, dietary substrates, and metabolite availability.

In summary, imbalance among Th17, Tfh17, Treg, and Tfr cells is not merely an isolated change in T-cell proportions, but a systemic immune state jointly shaped by the microbiota, metabolites, DCs, B cells, and synovial inflammation. Once established, this state can promote inflammatory cytokine release, enhance follicular responses, and weaken immune tolerance, thereby providing a foundation for autoantibody generation and local amplification of joint inflammation.

### 4.3. B Cells, IgA/IgG Switching, and ACPA Generation Link Mucosal Immunity with Autoantibody Responses

One of the most characteristic immunological events in RA is autoantibody generation, particularly the production of ACPA and RF. ACPA can be detected many years before the onset of clinical synovitis, suggesting that RA does not begin with local joint inflammation alone, but may undergo a prolonged phase of extra-articular autoimmune priming. The mucosal-origin hypothesis proposes that inflammation, microecological disturbance, protein citrullination, and local B-cell activation in barrier tissues such as the lung, oral cavity, and gut may jointly induce autoantibody production and subsequently promote the development of clinical RA through epitope spreading, class switching, and systemic dissemination [82].

IgA autoantibodies are particularly noteworthy in this process. IgA is the predominant antibody class in mucosal immunity, and the presence of IgA ACPA or secretory ACPA suggests that RA-related autoimmune responses may carry a mucosal imprint. Lung-related studies have shown that early ACPA-positive patients with RA may exhibit structural lung abnormalities and local antibody enrichment, supporting the involvement of mucosal sites in the initiation of RA-related autoimmunity [83]. Studies of the lung and oral cavity provide important reference points for the mucosal-origin hypothesis of RA, while the gut represents one of the key barrier sites deserving focused discussion because of its enormous antigenic load, strong IgA-inducing capacity, microbiota-dependent follicular responses, and continuous barrier exposure.

In the context of dysbiosis, barrier disruption, and enhanced Tfh/Tfh17 help, local B cells may undergo IgA class switching and participate in systemic autoantibody networks through circulatory migration, antigenic cross-reactivity, or bystander activation. Tfr insufficiency may further weaken the restriction of abnormal follicular responses, making B cells more prone to aberrant activation, class switching, and epitope spreading. Thus, the gut microbiota does not necessarily directly induce all RA-related autoantibodies, but may provide an immune ecological niche for autoantibody clone selection, expansion, and systemic dissemination.

Existing studies have linked specific gut strains to the generation of RA-related autoantibodies. Clonal IgA and IgG autoantibodies isolated from individuals at high risk for RA were found to recognize specific *Subdoligranulum* strains. These strains can not only be cross-recognized by RA-related autoantibodies but also activate RA-associated T cells and induce arthritis-like phenotypes in mice [17]. This finding suggests that IgA/IgG autoantibodies derived from some RA high-risk individuals may participate in the transition from mucosal autoimmunity to arthritis-like phenotypes through cross-recognition of gut bacterial antigens. In addition, *Eggerthella lenta* can enhance preclinical autoantibody generation in humanized arthritis models and induce metabolically aged immune changes and more severe arthritic phenotypes [16]. These studies indicate that the gut microbiota may participate in the formation of autoantibody networks through mechanisms such as molecular mimicry, cross-reactivity, metabolic remodeling, or bystander activation.

Therefore, B-cell and autoantibody responses can be summarized at three levels. First, the intestinal mucosa provides an environment for antigen exposure, B-cell activation, and IgA class switching. Second, Tfh/Tfh17 expansion and Tfr insufficiency enhance aberrant B-cell help, promoting IgA/IgG autoantibody responses and epitope spreading. Third, antigens from certain microbial strains may participate in the clonal expansion of ACPA/RF-related B cells through cross-recognition or bystander activation. Compared with the traditional model of local synovial autoantibody generation, the mucosal–gut perspective better explains why RA-related autoimmunity can emerge years before arthritis and why it is jointly associated with abnormalities in the oral, pulmonary, and intestinal microbiota.

### 4.4. Immune-Cell Migration, Synovial Reactivation, and Joint Structural Damage

Whether gut immune signals ultimately translate into clinical RA depends on two sequential processes. The first is whether preactivated T cells, B cells, plasma cells, autoantibodies, inflammatory mediators, microbial products, and metabolites generated in the gut can enter the systemic circulation and affect distal tissues. The second is whether the local synovium provides a pathological microenvironment capable of receiving, amplifying, and sustaining these signals. In other words, the gut does not induce arthritis in isolation; rather, mucosal immune abnormalities are converted into local joint inflammation through circulatory dissemination and synovial reactivation.

In this process, immune-cell migration and synovial repositioning are key intermediate steps. While inducing T-cell differentiation, intestinal DCs can confer specific homing and migratory properties. Tfh/Tfh17-like cells, Th17 cells, and aberrantly activated B cells can then participate in systemic immune responses through circulation. When these cells or their products enter the synovium, local inflammatory cytokines, chemokines, vascular activation, and antigen-presenting cells can further promote their reactivation. In RA synovium, interactions between DCs and T cells can support Th17 polarization, and synovial CD1c^+^ DCs can produce Th17-promoting cytokines such as IL-1β, IL-6, and IL-23 [74], providing joint-level evidence for a continuous process of “initial activation in the gut–peripheral expansion–local reactivation in the joint.”

FLS, macrophages, and osteoclasts are the core effector cells in this local amplification process. Microbiota-derived signals can act on local joint cells through two major routes. The first route involves microbial products or vesicles crossing the barrier, entering the circulation, and directly activating innate immunity within the joint. Studies of Fusobacterium nucleatum have shown that its FadA-containing outer membrane vesicles can aggravate RA-like inflammation and act on synovial macrophage-related inflammatory pathways [15]. This indicates that, under conditions of barrier injury, microbial structural components may not only serve as local intestinal inflammatory stimuli but may also influence synovial macrophages and local inflammatory amplification through circulatory dissemination. Consistent with this, circulating bacterial DNA and markers of intestinal barrier damage or bacterial translocation, such as intestinal fatty acid-binding protein, are higher in patients with RA than in healthy controls, suggesting that gut-derived microbial components have the opportunity to enter the systemic circulation and participate in distal joint inflammation [33]. These microbial products can activate macrophages and FLS through pattern-recognition receptors such as TLR4, which is upregulated in macrophages and synovial fibroblasts from patients with RA and is considered an important node linking microbial products, innate immunity, and synovitis [84]. The second route involves microbial metabolites reshaping joint effector cells through immune or non-immune pathways. SCFAs are the most representative example in this regard. Studies have shown that gut butyrate metabolism-related bacteria are associated with RA autoantibody generation and bone erosion [39], suggesting that the SCFA axis affects not only mucosal Treg/Breg responses but may also be linked to osteoclastogenesis and abnormal bone metabolism. Further studies have shown that SCFAs, particularly propionate and butyrate, can directly reprogram the metabolism of osteoclast precursors and inhibit core osteoclastogenic pathways such as TRAF6 and NFATc1, thereby increasing bone mass and alleviating inflammatory bone loss [85]. This indicates that gut-derived metabolites can influence RA not only indirectly through immunoregulatory mechanisms such as Treg/Breg induction, but also directly at the level of osteoimmunology by limiting osteoclast differentiation.

Beyond SCFAs, the connection between host nutrient metabolism and osteoimmunology also deserves attention. Studies have demonstrated that L-arginine metabolism can suppress TNFα-induced osteoclastogenesis in both human and mouse cells by altering the energy metabolism of inflammatory osteoclasts and promoting purine metabolism-related changes, thereby alleviating bone erosion in multiple arthritis models [86]. These findings suggest that bone erosion in RA is not determined solely by inflammatory cytokines, but is jointly regulated by inflammation, immunometabolism, microbiota-associated metabolites, and host nutrient metabolism.

**Figure 3:**
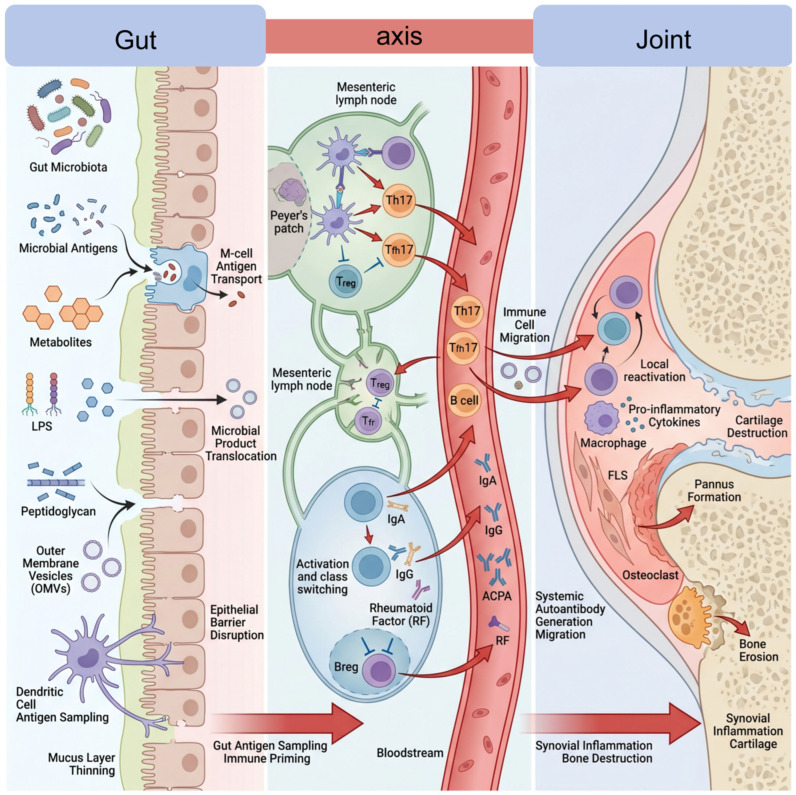
Gut microbial signals promote mucosal immune priming, systemic dissemination, and joint inflammation in rheumatoid arthritis.

In summary, the gut microbiota and its derivatives undergo mucosal immune initiation, systemic T/B-cell abnormalities, antibody generation, circulatory dissemination, and synovial reactivation, ultimately becoming amplified within the local pathological joint niche composed of FLS, macrophages, and osteoclasts and being converted into structural damage, as shown in Figure 3. This process explains why mucosal and systemic immune abnormalities can already be present before the onset of clinical arthritis in RA, and also suggests that targeting the gut microbiota, barrier function, metabolites, and immune-cell migration may provide a new theoretical basis for early intervention and precision treatment in RA.

## 5. Intervention Strategies Targeting the Gut Microbiota and Its Metabolites

Current RA treatment is based on treat-to-target strategies centered on MTX-based csDMARDs, bDMARDs, and tsDMARDs, whereas gut microecological interventions may serve as adjunctive strategies for disease prevention, therapeutic enhancement, response prediction, and stratified management of difficult-to-treat RA. As discussed in Section 2.3, the pretreatment gut microbiota may influence responses to MTX and DMARDs, while MTX itself can reshape metabolic pathways in multiple human-derived gut bacteria, indicating that RA therapy is not solely directed at host immunity but involves a bidirectional drug–microbiota–metabolite–immune response network. Therefore, this section discusses the translational potential and limitations of targeting the gut microbiota and its metabolites from five perspectives: diet and prebiotics, probiotics and next-generation live biotherapeutics, postbiotics and metabolite supplementation, barrier-restorative therapies, and FMT/virome-based/combined ecological therapies.

### 5.1. Diet and Prebiotics Reshape Protective Fermentative Ecology

Diet is the most fundamental, sustainable, and relatively safe approach for modulating the gut microbiota. Its core function is not simply to increase or decrease a particular microbial taxon, but to reshape functional ecology by altering nutrient-substrate availability, thereby influencing complex polysaccharide degradation, cross-feeding networks, SCFA production, bile acid transformation, and mucus layer homeostasis. For RA, the most important mechanistic basis of dietary intervention lies in the fermentation of dietary fiber, resistant starch, oligosaccharides, and plant polysaccharides by intestinal anaerobes into acetate, propionate, butyrate, and other SCFAs. These metabolites can promote Treg/Breg function, suppress Th17 responses, improve barrier integrity, and may directly act on FLS and osteoclasts, thereby reducing synovial inflammation and bone erosion. Clinical studies have shown that short-term high-fiber intervention increases serum SCFA levels in patients with RA and is accompanied by reductions in some inflammatory mediators [87]. High-fiber multigrain supplementation has also been reported to improve DAS28, morning stiffness, pain, and inflammatory/oxidative stress markers [88]. In addition, among individuals at high risk for RA, higher serum SCFA levels are associated with a lower risk of progression to clinical arthritis, suggesting that diet-derived SCFAs may not only influence inflammatory burden in established RA but also participate in risk modulation during the preclinical stage [89]. However, high-fiber or prebiotic interventions should not be simplistically regarded as universally anti-inflammatory strategies. In the context of colonization by specific RA-derived *Prevotella copri* strains, a high-fiber diet may synergize with *Prevotella copri* and instead aggravate experimental arthritis by enhancing intestinal inflammation [36]. This indicates that the immunological consequences of dietary substrates depend on the pre-existing microbial niche. When butyrate-producing bacteria, *Bacteroides*, *Bifidobacterium*, and cross-feeding networks are relatively intact, fiber is more likely to be directed toward SCFA production and barrier homeostasis. Conversely, in the presence of pro-inflammatory Prevotella lineages, expansion of facultative anaerobes, or pre-existing mucosal barrier disruption, the same substrates may be redirected toward pro-inflammatory metabolism, mucus layer disturbance, or increased antigen exposure. Therefore, dietary intervention strategies should be stratified according to disease stage, baseline microbiota structure, specific strain lineages, SCFA levels, and barrier-related indicators.

Prebiotic strategies can be further classified into three categories. The first includes traditional fermentable fibers and oligosaccharides, such as inulin, fructo-oligosaccharides, galacto-oligosaccharides, and resistant starch, which mainly act by increasing potential SCFA-associated taxa, including *Bifidobacterium*, *Faecalibacterium*, *Roseburia*, and *Anaerostipes* [90]. The second category includes plant polysaccharides or food–medicine homologous polysaccharides with defined structural features. Their advantage lies in their ability to serve as substrates for complex polysaccharide utilization loci and glycoside hydrolase networks, thereby inducing specific cross-feeding ecologies [91]. However, their limitations include substantial structural heterogeneity, difficulty in batch standardization, and marked differences in metabolic outputs across distinct microbiota backgrounds. The third category is personalized prebiotics [92], which involves selecting substrates based on a patient’s baseline metagenome and metabolome to restore deficient functional pathways. For example, in individuals with reduced butyrate-producing capacity, substrates that promote precursor–butyrate cross-feeding may be selected; in those with disordered bile acid metabolism, design may incorporate BSH-active bacteria and bile acid profiles; and in individuals with excessive mucus degradation, short-term simple increases in readily fermentable substrates should be avoided to prevent further mucus layer disturbance.

Therefore, the rational positioning of diet and prebiotics in RA should be as ecological substrate therapy. Their advantages include high safety, long-term feasibility, potential synergy with DMARDs, and possible application in primary or secondary prevention among RA high-risk populations. Their limitations include slow onset of action, substantial interindividual variability, dependence on baseline microbiota structure, and a lack of sufficiently large randomized controlled trials with long-term follow-up and endpoints such as imaging-defined bone damage or sustained remission.

### 5.2. Probiotics and Next-Generation Live Biotherapeutics Restore Missing Functions

The rationale for probiotic intervention is to restore protective ecological functions that are deficient in patients with RA through exogenous supplementation of live microorganisms with immunomodulatory capacity. Previous clinical trials in RA have mainly focused on traditional probiotics such as *Lactobacillus* and *Bifidobacterium*. Randomized, double-blind, placebo-controlled studies have shown that 8 weeks of probiotic supplementation can improve DAS28 and some metabolic parameters [93]. Synbiotic supplementation has also been reported to improve hs-CRP, DAS28, VAS, insulin resistance, and oxidative stress-related markers [94]. These studies provide preliminary clinical evidence supporting probiotics as adjunctive therapy for RA. However, the overall sample sizes are small, strain compositions are inconsistent, follow-up periods are short, and most studies do not simultaneously verify strain colonization, measure metabolites, or analyze immune-cell subsets. Therefore, it remains difficult to determine whether the observed effects are attributable to the strains themselves, placebo effects, dietary changes, or interactions with conventional pharmacological therapy. Mechanistically, traditional probiotics may enhance epithelial tight junctions, promote mucus layer homeostasis, increase SCFAs or lactate cross-feeding, reduce LPS burden, regulate DC maturation, and restore the Treg/Th17 balance. As noted above, *Lactobacillus casei* CCFM1074 alleviates arthritis in CIA models by regulating the gut microbiota, metabolites, and Treg/Th17 balance [81], suggesting that traditional probiotics may act not only through colonization but also through short-term metabolic outputs, ecological competition, and immune education. Systematic reviews also suggest that *Lactobacillus casei*-related strains are among the more promising candidates in RA probiotic research, although at present they should still be regarded as adjunctive interventions rather than standalone therapies [95].

Compared with traditional probiotics, next-generation live biotherapeutics place greater emphasis on disease-relevant functions and strain specificity rather than selection based only on commonly used probiotic genera. *Prevotella histicola* is one of the most representative candidates in RA research [37]. Other candidates may include specific *Bacteroides*, *Bifidobacterium*, *Faecalibacterium*, *Anaerostipes*, *Roseburia*, or rationally designed synthetic microbial consortia [75]. Their advantage lies in more clearly defined mechanisms, allowing design around SCFA production, bile acid transformation, tryptophan–indole metabolism, barrier repair, or induction of immune tolerance. Their limitations include difficulty in cultivation and formulation, poor stability of anaerobes, and substantial colonization resistance among patients. Therefore, future RA live biotherapeutics should establish a screening framework integrating “strain genome–metabolic function–receptor pathway–target cell–clinical phenotype,” and should prioritize stratified trials in early RA, high-risk RA, or patients with poor DMARD responses but without severe immunosuppressive risk.

### 5.3. Postbiotics and Metabolite Supplementation Enable Functional Replacement Therapy

Postbiotics and metabolite supplementation represent a strategy that shifts from supplementing microbes to supplementing functions. According to the ISAPP consensus, postbiotics are preparations composed of inactivated microorganisms and/or their components that confer a health benefit on the host, differing from probiotics in that they do not require microbial viability [96]. This feature offers potential advantages in RA, because many patients receive immunosuppressive therapy and live microbial products may involve unstable colonization, infection risk, and batch variability. By contrast, postbiotics, bacterial components, extracellular vesicles, metabolites, or metabolite prodrugs are more amenable to standardization, dose control, and drug development.

Among metabolite supplementation strategies, SCFAs—especially butyrate and propionate—remain the most mature direction. SCFAs have protective effects in RA, but direct supplementation of free butyrate is limited by odor, gastrointestinal irritation, short half-life, and insufficient systemic exposure. Therefore, pharmacological development of metabolites has become an important direction. A serine-conjugated butyrate prodrug has been developed with high oral bioavailability and was shown to suppress autoimmune arthritis and neuroinflammation in mice [40]. Such studies suggest that future RA metabolite therapies may evolve into metabolite prodrugs with defined pharmacokinetics, tissue distribution, and immune targets. In addition to SCFAs, tryptophan-derived indole metabolites and bile acid derivatives are also important candidates. Specific indole derivatives such as IPA and IAA can stabilize Treg cells through AhR, regulate the Th17/Treg balance, and alleviate CIA, whereas indole itself may promote the IL-23/Th17 axis and autoantibody responses under certain inflammatory conditions [57,58]. Therefore, indole metabolite supplementation must adhere to molecular specificity and should not broadly regard all AhR ligands as protective factors. For bile acids, different molecules exert markedly distinct effects on FXR, TGR5, VDR, and RORγt [61,62,63]. Future studies need to clarify the specific molecules, doses, target cells, and long-term metabolic safety profiles.

Postbiotics may also include inactivated bacterial cells, bacterial extracellular vesicles, cell-wall components, and engineered metabolite delivery systems. Compared with live microorganisms, their advantages include improved safety and quality control; their disadvantages include the lack of self-amplification and niche-occupying capacity, which may necessitate repeated administration. In addition, some bacterial components themselves may activate TLR/NOD-like receptors and produce pro-inflammatory effects [97]. Therefore, when developing postbiotic therapies for RA, priority should be given to candidates with clearly defined anti-inflammatory receptor pathways, controllable dose–response relationships, and monitorable pharmacodynamic biomarkers, such as SCFA prodrugs, specific AhR ligands, TGR5 agonist-related metabolites, or microbiota-derived molecules that promote epithelial repair.

### 5.4. FMT, Virome-Based Intervention, and Combined Ecological Therapy Rebuild Microecological Homeostasis

FMT is a strategy for resetting the gut ecosystem. Its advantage lies in the ability to introduce multiple microbial species, metabolic pathways, and complex ecological interactions simultaneously, rather than a single strain or metabolite. Case reports in RA have shown improvements in DAS28, HAQ-DI, and RF after FMT in patients with refractory RA, suggesting potential therapeutic value [98]. Meanwhile, clinical trials of FMT in patients with RA who respond inadequately to MTX have been registered, with the aim of evaluating the efficacy and safety of FMT combined with MTX [99]. However, current evidence for FMT in RA remains extremely limited and does not support routine clinical application. Major concerns include substantial donor variability, insufficient graft standardization, unknown long-term safety, infection risk in immunosuppressed patients, uncertain colonization durability, and the possibility that FMT may simultaneously transfer pro-inflammatory strains, virome components, antibiotic resistance genes, or unknown metabolic functions.

The virome, particularly the gut bacteriophage community, is an emerging direction in RA microecological intervention. Studies have shown that gut bacteriophage communities in first-degree relatives at high risk for RA can diverge according to anti-CCP status and carry auxiliary metabolic genes associated with anti-CCP status. Recent paired-sibling studies in RA further indicate that patients with RA harbor an abnormal gut phageome, with some phages showing potential to induce autoimmunity [100]. In addition, gut virome studies in specific regional populations also suggest associations between virome alterations and autoimmune diseases [101]. These findings indicate that RA-associated gut ecological abnormalities involve not only the bacterial microbiome but also cross-kingdom interactions among bacteriophages, bacteria, and metabolic pathways. Phages may participate in RA pathology by lysing specific bacteria, altering bacterial competition, carrying auxiliary metabolic genes, or influencing bacterial antigen release. Virome-based intervention may take three forms. The first is targeted phage therapy, which uses specific phages to eliminate pro-inflammatory or pathogenic strains [102]. For example, future studies could explore whether specific pathogenic lineages of *Prevotella copri*, *F. nucleatum*, or *E. lenta*-related strains can be selectively inhibited. The second is fecal virome transplantation, which aims to remove bacteria as far as possible while retaining the virome to reshape bacterial ecology and metabolic function [103]. Theoretically, this strategy may carry a lower infection risk than complete FMT, but it remains at the preclinical and proof-of-concept stage in RA. The third is combined phage–probiotic therapy, in which phages are first used to reduce pro-inflammatory microbial niches, followed by supplementation with protective strains or prebiotics to promote restorative colonization [104]. Key challenges include narrow host range, rapid emergence of bacterial anti-phage resistance, phage immunogenicity, and ecological spillover effects. Therefore, virome-based intervention is currently more appropriate as a future research direction than as a near-term clinically deployable RA therapy.

In summary, RA intervention strategies targeting the gut microbiota and its metabolites should move from empirical supplementation toward mechanism-oriented and stratified treatment. Diet and prebiotics are suitable for long-term ecological modulation; probiotics and next-generation live biotherapeutics are suitable for restoring specific missing functions; postbiotics and metabolite prodrugs may enable controllable pharmacological intervention; barrier-restorative therapies may block sustained gut-derived inflammatory input; and FMT, virome-based intervention, and combined ecological therapies represent more powerful but also more cautious directions for ecological resetting. International consensus on microbiome testing further reminds us that clinical microbiome analysis requires standardized sample collection, sequencing analysis, report interpretation, and indication assessment, and that commercial microbiome test results should not be directly equated with treatment decisions [105]. Future high-quality studies should prioritize longitudinal, multi-omics, mechanism-embedded randomized trials that simultaneously evaluate microbial changes, metabolites, barrier function, immune-cell migration, and joint outcomes, thereby providing a more reliable and authoritative basis for determining whether gut microecological intervention can become part of precision RA treatment.

## 6. Discussion

In recent years, research on the gut microbiota in RA has gradually shifted from the early search for RA-characteristic taxa toward understanding how microbial functions at specific disease stages influence autoimmune initiation, inflammatory persistence, and therapeutic responses. The central argument of this review is that RA-associated gut microecological abnormalities should not be interpreted as a simple increase or decrease in a fixed genus or species, but rather as a dynamic functional ecosystem shaped jointly by genetic susceptibility, preclinical autoimmunity, overt synovitis, chronic inflammation, mucosal barrier status, and drug exposure. Microbiota instability in preclinical RA, *Prevotella copri* and other immunologically active strain signals in new-onset untreated RA, barrier damage and microbial product exposure in chronic RA, and the bidirectional relationship between post-treatment microbial remodeling and drug response collectively suggest that the gut microbiota is not an isolated accompanying phenomenon in RA pathogenesis, but may represent a key ecological variable involved in the continuous evolution of the disease [3,18,19,20,30].

This perspective helps explain the heterogeneity commonly observed in previous studies of RA and the gut microbiota. Microbial signals involving *Prevotella*, *Collinsella*, *Bacteroides*, *Eggerthella*, *Subdoligranulum*, and other taxa do not always show consistent directional changes across cohorts. This inconsistency may not be attributable solely to technical differences, but may also reflect differences in disease stage, host genetic background, dietary pattern, drug exposure, strain lineage, and functional pathways. Taking *Prevotella copri* as an example, species-level abundance alone is insufficient to explain its complex relationship with RA. Strain-level genomic features, mobile genetic elements, antigenic epitopes, and immunostimulatory capacity may have greater mechanistic significance than enrichment per se [7,24,25]. Therefore, future RA microecological research can resolve continuous evidence chains linking strain, functional gene, metabolite, receptor, target cell, and RA phenotype.

Microbiota-derived metabolites provide relatively clear mechanistic nodes connecting gut functional ecology with host immune effects. SCFAs, tryptophan-derived indole metabolites, bile acids, and succinate are not isolated metabolic end products, but rather the result of interactions among specific substrates, microbial enzymatic systems, and the host immune milieu. SCFAs, especially butyrate and propionate, can exert protective effects by promoting Treg/Breg responses, regulating AhR signaling, inhibiting the inflammatory phenotype of FLS, and limiting osteoclastogenesis [10,11,51,85]. Tryptophan-derived indole metabolites show more pronounced ligand specificity: IPA, IAA, and related metabolites can promote Treg homeostasis and suppress inflammation through AhR-related pathways, whereas indole itself may enhance Th17- and antibody-mediated arthritic responses under certain inflammatory contexts [55,56,57]. The bile acid axis further indicates that the microbiota affects not only local intestinal metabolism but also barrier function, T-cell differentiation, and joint inflammation through signals such as TGR5 and FXR [60,61,62,63]. By contrast, succinate is more closely associated with inflammatory metabolism and tissue injury, promoting macrophage activation, Th17 expansion, angiogenesis, and persistent synovial inflammation through SUCNR1/GPR91 [65,66,67]. These findings indicate that the significance of microbial metabolites in RA does not lie in a general anti-inflammatory or pro-inflammatory classification, but in the fact that their source, concentration, receptor engagement, target cells, and disease stage jointly determine their final effects.

From an immunological perspective, the key mechanism by which the gut microbiota influences RA is not merely “leaky gut” or the entry of microbial products into the circulation, but how intestinal mucosal immune signals are initiated, shaped, mobilized, and amplified within the joint. Intestinal antigen sampling, DC-mediated T-cell differentiation, imbalance between Th17/Tfh17 and Treg/Tfr cells, B-cell class switching, and expansion of IgA/IgG autoantibodies constitute important pathways through which gut-derived signals are converted into systemic autoimmunity [13,14,17,71,72,73,74,75,76]. Certain gut strains can be recognized by RA-related autoantibodies or induce T-cell and B-cell responses, suggesting that the gut may serve as one mucosal site for autoimmune clonal selection and expansion in RA [16,17]. However, this does not mean that all cases of RA originate in the gut, nor does it imply that the gut microbiota can explain the entirety of RA pathology. A more appropriate interpretation is that the gut, together with other mucosal sites such as the oral cavity and lung, forms a network for the extra-articular initiation and amplification of RA-related autoimmunity, whereas local joint effector cells, including FLS, macrophages, and osteoclasts, ultimately determine whether inflammation develops into persistent synovitis and structural damage.

In terms of clinical translation, this review emphasizes that gut microecological interventions should be positioned cautiously within the existing RA treatment framework. The cornerstone of current RA therapy remains treat-to-target management based on MTX-centered csDMARDs, bDMARDs, and tsDMARDs, and gut microbiota interventions should not be presented as alternatives. A more evidence-aligned positioning is as follows: in preclinical RA or high-risk populations, they may support risk identification and preventive ecological modulation; in new-onset or active RA, they may assist in predicting MTX or DMARD responses; in patients with poor treatment responses or unstable remission, they may contribute to therapeutic sensitization, barrier improvement, and reduction in inflammatory input; and in long-term management, they may serve as part of dietary, prebiotic, metabolite-supplementation, and lifestyle interventions [18,19,20,30,87,88,89].

Despite the rapid development of studies linking the gut microbiota with RA, important limitations remain. First, most human studies are still cross-sectional, making it difficult to distinguish whether microbial alterations are causal contributors to RA, consequences of inflammation, or results of drug exposure. Second, although animal models provide causal evidence, CIA, K/BxN, and other models cannot fully recapitulate the genetic background, clinical heterogeneity, and long-term medication environment of human RA. Third, many studies remain at the genus or species level and lack simultaneous integration of strain-level resolution, functional gene expression, measured metabolites, and host immune phenotypes [105]. Fourth, most microecological intervention studies are limited by small sample sizes, short follow-up duration, heterogeneous endpoints, and a lack of outcomes related to imaging-defined structural damage or sustained remission. Future research can advance in three directions. First, longitudinal multi-omics cohorts should be established across RA high-risk individuals, new-onset untreated RA, active RA, remission-stage RA, and difficult-to-treat RA, integrating metagenomics, metatranscriptomics, metabolomics, barrier markers, immune-cell profiling, and clinical outcomes to identify truly stage-specific microbial functional biomarkers. Second, mechanism-embedded studies should be conducted using strain isolation and culture, germ-free or colonized animal models, FMT, stable isotope tracing, and targeted metabolite supplementation to validate causal chains linking “specific strains/enzymatic systems–metabolites–receptors–target cells–RA phenotypes.” Third, stratified randomized controlled trials should be designed by selecting intervention populations according to baseline microbiota structure, metabolite levels, barrier status, disease stage, and medication background, and by adopting composite endpoints including DAS28, ACR responses, imaging progression, relapse rate, remission maintenance, safety, and restoration of microbial functions. These steps are required before microbiome findings can become predictable and clinically actionable.

Taken together, RA-associated gut microbiota should be understood not as a fixed taxonomic signature, but as a stage-dependent functional ecosystem embedded within the gut–joint axis. Its pathogenic or protective effects are determined by the interaction among strain-level variation, ecological niche competition, barrier integrity, microbial metabolic outputs, and host immune context. Microbiota-derived metabolites provide key mechanistic links through which intestinal ecological changes are translated into mucosal immune activation, systemic autoimmunity, immune-cell trafficking, synovial reactivation, and structural joint damage. Therefore, the future of RA microbiome research should move beyond association-based profiling toward longitudinal, strain-resolved, multi-omics and mechanism-driven studies that define which microbial functions act at which disease stages, through which metabolites and immune pathways, and in which patient subgroups. Within this framework, microbiota-targeted strategies should be positioned as precision adjunctive approaches to current RA management, with potential value in risk stratification, therapeutic response prediction, treatment sensitization, and long-term disease control, rather than as replacements for established immunomodulatory therapies.

## Data Availability

No new data were created or analyzed in this study. Data sharing is not applicable to this article.

## Abbreviations

The following abbreviations are used in this manuscript:

5-HIAA	5-Hydroxyindole-3-acetic acid
ACPA	Anti-citrullinated protein antibodies
ACR	American College of Rheumatology
AhR	Aryl hydrocarbon receptor
anti-CCP	Anti-cyclic citrullinated peptide antibodies
AUC	Area under the curve
bDMARDs	Biologic disease-modifying antirheumatic drugs
Breg	Regulatory B cell
BSH	Bile salt hydrolase
CA	Cholic acid
CCL	CC chemokine ligand
CCR	CC chemokine receptor
CD	Cluster of differentiation
CIA	Collagen-induced arthritis
CRP	C-reactive protein
csDMARDs	Conventional synthetic disease-modifying antirheumatic drugs
CXCL	CXC chemokine ligand
DAS28	Disease Activity Score in 28 joints
DC	Dendritic cell
DCA	Deoxycholic acid
DMARDs	Disease-modifying antirheumatic drugs
FLS	Fibroblast-like synoviocytes
FMT	Fecal microbiota transplantation
FOXK1	Forkhead box protein K1
Foxp3	Forkhead box protein P3
FXR	Farnesoid X receptor
GPBAR1/TGR5	G protein-coupled bile acid receptor 1
GPR	G protein-coupled receptor
HAQ-DI	Health Assessment Questionnaire-Disability Index
HDAC	Histone deacetylase
HIF-1α	Hypoxia-inducible factor 1-alpha
HLA	Human leukocyte antigen
H4R	Histamine H4 receptor
hs-CRP	High-sensitivity C-reactive protein
IAA	Indole-3-acetic acid
IAld	Indole-3-aldehyde
IDO1	Indoleamine 2,3-dioxygenase 1
IFN	Interferon
Ig	Immunoglobulin
IL	Interleukin
ILC	Innate lymphoid cell
IPA	Indole-3-propionic acid
ISAPP	International Scientific Association for Probiotics and Prebiotics
LCA	Lithocholic acid
LPS	Lipopolysaccharide
MTX	Methotrexate
NF-κB	Nuclear factor kappa B
NFATc1	Nuclear factor of activated T-cells, cytoplasmic 1
NOD	Nucleotide-binding oligomerization domain
PRR	Pattern recognition receptor
RA	Rheumatoid arthritis
RF	Rheumatoid factor
RORγt	RAR-related orphan receptor gamma t
SCFAs	Short-chain fatty acids
SIRT5	Sirtuin 5
SUCNR1	Succinate receptor 1
TAZ	Transcriptional coactivator with PDZ-binding motif
TDO	Tryptophan 2,3-dioxygenase
Tfh	T follicular helper cells
Tfh17	IL-17-producing T follicular helper cells
Tfr	T follicular regulatory cells
TGF-β	Transforming growth factor beta
Th1	Type 1 T helper cells
Th17	Type 17 T helper cells
TLR	Toll-like receptor
TMAO	Trimethylamine N-oxide
TNF-α	Tumor necrosis factor-alpha
TRAF6	TNF receptor associated factor 6
Treg	Regulatory T cell
tsDMARDs	Targeted synthetic disease-modifying antirheumatic drugs
UDCA	Ursodeoxycholic acid
VAS	Visual Analog Scale
VDR	Vitamin D receptor
VEGF	Vascular endothelial growth factor
YB-1	Y-box binding protein 1
7α-HSDH	7α-hydroxysteroid dehydrogenase
